# Measurement and evaluation of the coupling degree between National Fitness and National Health in the Yangtze River Economic Belt region of China

**DOI:** 10.3389/fpubh.2023.1209854

**Published:** 2023-09-22

**Authors:** Changwen Cai, Songzhong Ye

**Affiliations:** Sports Industry Development Research Center, Fujian Jiangxia University, Fuzhou, China

**Keywords:** national fitness, national health, coupling degree, coupling coordination degree, entropy method

## Abstract

Clarifying the integrated development mechanism of the National Fitness Program and National Health Program has positive and practical significance for the efficient implementation of the healthy China strategy. In order to analyze and study the integrated development mechanism of the National Fitness Program and National Health Program, we established a coupling-coordination measurement model. Based on two dimensions of space and time, we first analyzed the coupling degree between National Fitness System and National Health System in the Yangtze River Economic Belt region of China from 2016 to 2020, and studied and analyzed the coupling mechanism between the two. The research results show that the National Fitness Program and National Health Program have a development model of mutual promotion and mutual benefit. The National Fitness Program promotes the National Health Program by improving the fitness environment, increasing fitness participation, and health investment. The National Health Program optimizes environmental construction, health education, and healthy populations, thereby driving national fitness. Overall, there is stable development and balanced promotion in the integration of the National Fitness Program and National Health Program. In addition, the integrated development of the National Fitness Program and National Health Program is positively affected by economic level and regional marketization. The coupling- coordination model adopted in this study has practical value in measuring the coupling degree between the National Fitness System and National Health System. The results of this study also provide an important reference basis for the integrated and coordinated development of the National Fitness Program and National Health Program and the implementation of the Healthy China strategy.

## 1. Introduction

As the world economy develops and society progresses, there is an increasing demand and attention to health. National fitness and national health have become important factors in social development and the improvement of people’s well-being. The integrated development of national fitness and national health aligns with the global trend of health development. More and more countries and regions worldwide are treating national fitness and national health as important strategies, promoting people’s health through measures such as providing fitness facilities, promoting health education, and strengthening health management. In response to this, General Secretary Xi Jinping of China has repeatedly emphasized the advocacy of a healthy and civilized lifestyle, the improvement of the health literacy of the entire population, and the deep integration of national fitness and national health during the period of 2016–2022. Meanwhile, the Chinese government has issued the “National Fitness Plan (2021–2025)” and the “Sports Law of the People’s Republic of China,” which propose the implementation of a national fitness strategy, the establishment of a public service system for national fitness, and the encouragement and support of citizens’ participation in fitness activities to promote the deep integration of national fitness and national health (1, 2). The essence of the integration of the National Fitness Program and National Health Program is the exploration of promoting health, and their common goal is to address the current health issues faced by China and promote the implementation of the Healthy China strategy. Therefore, in the context of reality and policy, it is particularly important to measure and evaluate the degree of integration of national fitness and national health. Calculating and evaluating the coupling degree of National Fitness Program and National Health Program can provide decision support and guidance for the government and relevant departments, promote the rational allocation and optimal utilization of resources, and play an important guiding role in promoting the deep integration of the National Fitness Program and National Health Program and improving the health level of the population.

So far, the academic research based on the relationship between the National Fitness Program and National Health Program mainly has focused on the integration of the two, including the following three aspects. (1) In terms of the connotation of the integration of the National Fitness Program and National Health Program, many scholars have started their research on the integration of the two systems by tracing the source of concepts. For examples, Lu (3) believes that the integration of the two is a process of mutual penetration and integration in a wider range, higher level, and deeper degree. Qiu (4) proposes that connotation integration is led by the integration of physical education and medicine, guided by health goals, and fitness as a means and approach. Lan (5) believes that National Fitness Program should take National Health Program as the goal and direction, and National Health Program should take National Fitness Program as an important way and means to form a new pattern of development. Scholars have deeply studied the connotation definition of National Fitness and National Health, but there is a lack of research on the mutual integration and impact mechanism of the two systems. This connotation can provide a more accurate indicator system construction for the subsequent mutual impact mechanism of the two systems.

(2) On the integration path of the National Fitness Program and National Health Program, much progress has been made. For examples, Lu (3) formulated a path choice from historical logic and realistic logic, and explored the implementation of the National Fitness Program and National Health Program from multiple perspectives. Shen (6) studied and analyzed the practical difficulties of the integration of the National Fitness Program and National Health Program, and proposed to implement the path from the six integration dimensions of “department, policy, talent, organization, re-sources, and industry” to promote deep integration. You (7) examined the integration path of the two from a policy perspective and proposed a policy process of deep integration of the two in the new era. Li (8) analyzed the challenges and practical dilemmas faced by the integration of the National Fitness Program and National Health Program, and proposed specific integration paths: concept first, physical reform, removing policy obstacles, establishing policies and regulations, and promoting the cultivation of versatile talents. The above literature has conducted in-depth analysis of the integration path of the National Fitness Program and National Health Program, with fruitful results, while ignoring that the two will be affected to varying degrees in various aspects in the integration path.

(3) In terms of the mechanism of integrating the National Fitness Program and National Health Program, Lu (3) believes that the institutional mechanism for integrating the National Fitness Program and National Health Program is a way to connect different entities involved in integration, enabling them to coordinate and function, achieve integration goals and effects, and it is a dynamic approach. Zufeya (9) believes that the mechanism of integrating the National Fitness Program and National Health Program is a strategic system of multi-departmental collaboration and comprehensive governance, where the integration process is achieved through the linkage of macro decision-making, meso-level management, and individual participation. Han (10) views the synergistic mechanism of integrating the National Fitness Program and National Health Program as a coordinated operating method of multiple stakeholders, where they promote the deep integration of the National Fitness Program and National Health Program through mutual cooperation and continuous development. Although scholars have different interpretations of the mechanism of integrating the National Fitness Program and National Health Program, one point has reached a consensus, namely that the mechanism involves coordination and cooperation among different entities or multiple departments to facilitate the dynamic development of their integration. In recent years, scholars have explored the integration of the National Fitness System or the National Health System with other systems. Qian (11) proposed a path for coupled development based on the coupling development mechanism of the National Fitness Program and sports industry. Qi (12) analyzed the coupling mechanism between the National Health Program and an overall well-off society from three levels: goals, values, and energy. Although the above literature involves research on fusion mechanisms, it is limited to qualitative research and lacks a specific indicator system, namely quantitative research using entropy method and coupling coefficient model to establish mathematical models.

In general, scholars have achieved fruitful results in the research on the integration relationship between the National Fitness Program and overall health, and have conducted well. They have analyzed and explored the integration relationship between the two from multiple perspectives of integration. However, there are two problems in current relevant research: first, the research has remained theoretical research on the National Fitness Program and National Health Program, and there is a lack of data exploration and verification of the integration relationship between the National Fitness Program and overall health. The coupling relationship between the two will affect the progress and effectiveness of comprehensive fitness and the achievement of the goal of national health. The second is that the scope of research is large, the theoretical coverage is broad, involving the entire national level, and there is a lack of specific comparative analysis of representative regions and different provinces and cities. Therefore, in the context of the healthy China strategy, it is particularly important to measure and evaluate the integration of the National Fitness Program and National Health Program through a combination of qualitative and quantitative research. Based on the analysis of the coupling effect between the National Fitness Program and National Health Program in various aspects, this paper constructs a model based on the two dimensions of space and time, analyzes the coupling and coordination relationship, and verifies the integration mechanism between the two. The research results of this paper have positive practical significance for achieving the efficient implementation of the healthy China strategy.

## 2. The integration mechanism of the National Fitness and National Health program

### 2.1. Dimensional analysis of the National Fitness System

Since the State Council promulgated the “Regulations on National Fitness” and the “National Fitness Plan (2011–2015),” the National Fitness Program has been integrated into the overall planning of economic and social development of governments at all levels. After continuous efforts in recent years, the “big group” work pattern of “government led, department coordinated, and the entire society participated” has basically taken shape, and the effect has gradually become evident. Wei (13) regarded the National Fitness System as a structural system composed of government departments, market departments, and “nonprofit departments,” that is, “third departments.” Zhou (14) believes that the National Fitness service system is a social sports fitness guarantee system that can effectively meet people’s growing demand for physical fitness, effectively guarantee the active participation of the people in physical fitness activities, promote the general enhancement of national physique, and have diverse forms, rich content, sound mechanisms, effective operation, and actively integrate into the social service system under the conditions of socialist market economy. Dong (15) believes that the National Fitness System has service, security, and management functions. It is not only a content element system, but also a hierarchical element system, and more importantly, a “relationship” system. Luo (16) believes that the National Fitness service system is a combination of all soft and hard technologies used in the process of providing sports fitness services to meet the mutual benefits of service recipients. The models are divided into public service models and market service models. Xiao (17) believes that the National Fitness service system is an organic whole composed of elements that meet the needs of national fitness, including nine subsystems. The above viewpoints are also helpful for understanding and grasping the National Fitness system, and all have some limitations to some extent. In recent years, there has been less research on the coupling and coordination of national fitness. Feng (18) selected 17 first level indicators and 38 s level indicators from four levels of integration subjects, integration goals, integration conditions, and integration approaches, and constructed a path indicator system for the deep integration of the “National Fitness” and “National Health.” Therefore, based on the analysis of scholars on the national fitness system, combined with the analysis of indicators of the National Fitness System, this paper summarizes and refines the National Fitness System into three dimensions: the national health environment, national fitness participation, and national health investment.

### 2.2. Dimensional analysis of National Health System

The connotation of a Healthy China strategy is not only to ensure the health of the whole people, but also to ensure the overall health of the healthy environment, the healthy economy, and the healthy society. National health is also one of the main indicators to ensure the construction of a healthy city. The WHO defines a healthy city as an organic combination of healthy people, a healthy society, and a healthy environment. It has formulated 3 health indicators, 8 social indicators, 7 health service indicators, and 14 environmental indicators for a healthy city. In 2018, the China Health Commission released the “China Healthy City Evaluation Indicator System (2018 Version)” (19), which constructs a first level indicator from five dimensions: healthy environment, healthy society, health services, healthy people, and health culture, including 20 s level indicators and 42 third level indicators. Therefore, starting from the perspective of “Four in One” health, this paper summarizes and refines five dimensions based on the indicators of a healthy urban system, simplifies the model, and summarizes the national health system into three dimensions: health environment construction, health education promotion, and healthy population development. Among them, the construction of a healthy environment is the foundation of national health, the promotion of health education is the guarantee of national health, and the development of healthy populations is the goal of national health.

### 2.3. Analysis of the relationship between the National Fitness System and National Health System

The concept of coupling originates from physics and refers to the existence of a relationship of mutual influence and interaction between two or more systems. In terms of the relationship between national fitness and national health, the two have similarities in service targets, convergence in core goals, and overlap in means and methods. Therefore, it is necessary to explore and analyze the integration relationship and mechanism between the two before building the model.

#### 2.3.1. The relationship between national fitness and fitness environment, fitness participation, and health investment

The optimization of the national fitness environment can improve the development of the National Health System. The quality of the national fitness environment and the construction of the fitness environment are dialectically unified and complement each other. High environmental quality can greatly promote fitness activities. Conversely, low environmental quality is not conducive to the formation of residents’ fitness habits, it is difficult to form a good fitness atmosphere and crowd participation rate, and environmental construction will also lag behind. Therefore, forming an active fitness atmosphere, rich sports life, strong fitness awareness, and a good fitness ecological environment can improve the quality of national fitness, promote the construction of a healthy environment, and promote the completion of the national health system. Starting from the “hard environment,” increasing the availability and use of national fitness venues, facilities, and equipment, and popularizing the group fitness effect of community fitness can greatly improve fitness habits, promote the construction of a healthy environment, and further promote and improve the national health system.

The quality and quantity of participation in national fitness can improve the over-all health system. Constantly improve the public service system for national fitness, ensure that everyone enjoys basic sports fitness services, and consolidate the basic conditions for national fitness to promote national health (20). The strategic theme of the “Healthy China 2030” Planning Outline mentions “promoting a healthy lifestyle,” and fitness for all can be seen as a scientific, healthy, and effective healthy lifestyle for all. Therefore, on the premise of achieving the goal of achieving national health, we call on the people of the whole country to participate in physical exercise and improve the quality and quantity of national fitness participation. The fundamental goal is to strengthen the people’s physique, improve their health level, and then improve the national health system to achieve national health.

The volume ratio of national health investment can expand the National Health System. The health level of a country or region is usually measured by health input, which is reflected by human, material, and financial indicators of the health industry such as the total number of professional health personnel, the total number of medical and health institutions, and the total amount of health expenses (21).

#### 2.3.2. The relationship between national health and environmental construction, health education, and healthy populations

The construction of a healthy environment is the basic condition for promoting the development of national fitness. The development of national fitness needs to be supported by a good sports ecological environment. Through the construction and improvement of a healthy environment, such as increasing forest coverage and reducing smog days, more people can participate in physical exercise and expand the demand for sports market. National fitness has a high demand for healthy environmental conditions. Therefore, it is possible to start with managing the broken health environment, protecting and strengthening a good health environment, optimizing the construction of a healthy environment, and ultimately promoting the development of national fitness.

The promotion of health education is a key factor in improving the awareness and needs of national fitness. With the increasing satisfaction of material pursuits, more and more people are paying attention to health education, not only physical health, but also psychological and social health. When the awareness of national fitness is constantly strengthened and demanded, it will be beneficial to expand the fitness community and promote the development of national fitness.

The development of healthy people is an important guarantee for stabilizing the National Fitness System. The extension of life expectancy *per capita* is bound to bring about an aging population, and the increase of the aging population will bring more health problems for the older adult. Developing healthy exercise for the older adult is particularly important. The youth group is the main group participating in physical exercise, mainly ensuring that the youth group exercises in a good healthy environment, and ensuring the stability and improvement of group development, in order to stabilize and ensure the national fitness system.

**Figure 1:**
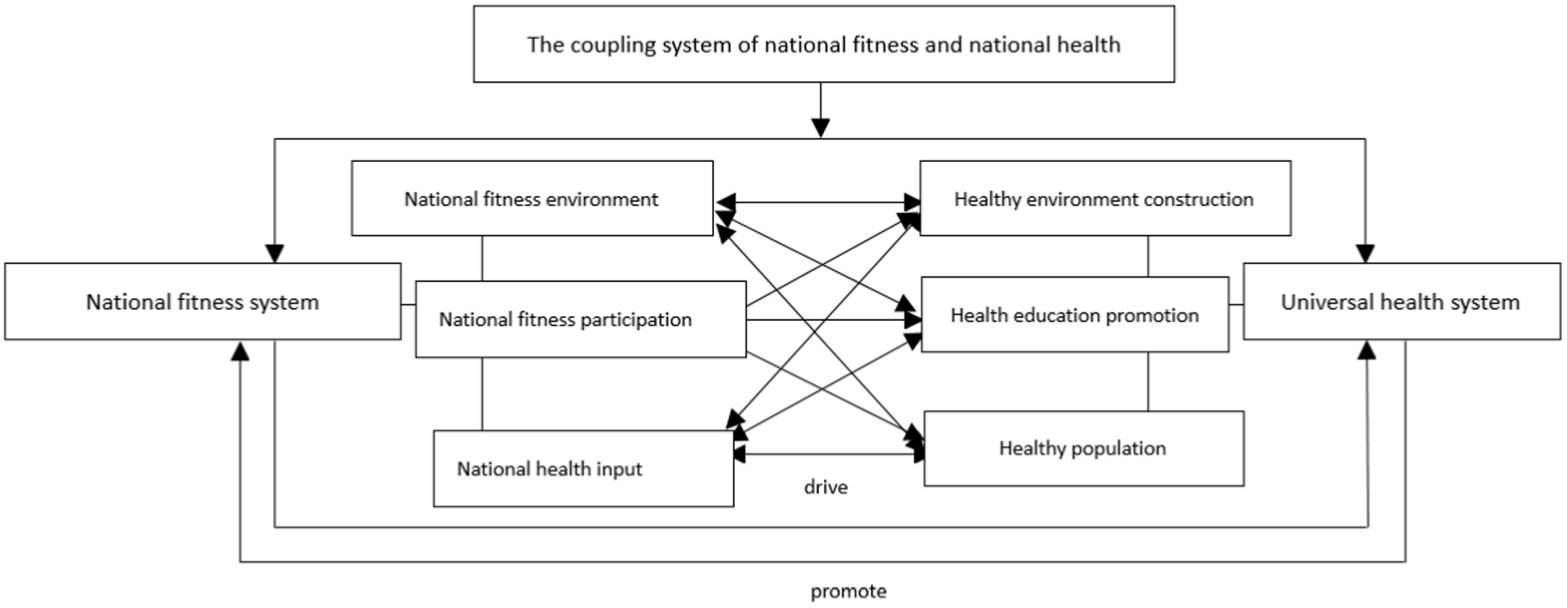
Mechanism model of coupling effect between the National Fitness Program and National Health Program.

Based on the above analysis and discussion, the coupling mechanism model of the National Fitness Program and National Health program is shown in Figure 1. The analysis shows that the goal and ultimate guidance of national fitness is national health, and the important way and means of national health is national fitness.

## 3. Research methods, indicator systems, and data sources

### 3.1. Research methods

#### 3.1.1. Dimensionless processing of data

Dimensionless processing can eliminate dimensional differences between variables, enhancing the stability and reliability of data analysis, improving the performance and effectiveness of models, reducing computational complexity, accelerating algorithm convergence, and enhancing the effectiveness of feature selection. The National Fitness System and National Health System are composed of several indicators. In order to unify the dimensions of each indicator, the raw data is standardized before data analysis. The calculation formula is as follows (22, 23):


(1) $$y_{ij}=\frac{x_{ij}-\min x_{ij}\;}{\max\;x_{ij}-\min x_{ij}}$$

(2) $$y_{ij}=\frac{\max\;x_{ij}-x_{ij}\;}{\max\;x_{ij}-\min x_{ij}}$$

Equation (1) is the normalization formula for positive indicators and Equation (2) is the normalization formula for negative indicators. Where *x_ij_* means the original data of the *j*-th indicator of a province or municipality in year *i*, and *y_ij_* means the standardized data of the j-th indicator of a province or municipality in year *i*. min *x_ij_* is the minimum value of the *i*-th indicator and max *x_ij_* is the maximum value of the *i*-th indicator. The data of the dimensionless processing has the value of 0, which will interfere and affect the later calculation, so the data is panned. The calculation formula is as follows:


(3) $$y\prime_{ij}=y_{ij}+\alpha$$

The α in Equation (3) is the translation amplitude. In this article, α = 0.01 is chosen to reduce the impact of panning data.

#### 3.1.2. Entropy method

The advantages of the entropy method include: the absence of subjective weight setting, consideration of the correlation between indicators, strong flexibility and adaptability, easily interpretable and understandable results, applicability to multi-objective decision-making, as well as flexibility and adjustability. These features make the entropy method a commonly used multi-criteria evaluation approach that finds wide application in various fields and scenarios. Therefore, in this paper the entropy method is used to measure the index weights of national fitness and national health. The specific process is as follows (24–26):

Step 1: Homogeneous metrics are quantified. Measure the share of the *i*-th year of the *j*-th indicator. The formula is as follows:


(4) $$p_{ij}=\frac{y_{ij}}{\sum_{i=1}^{m}y_{ij}}$$

Where *p_ij_* refers to the *i*-th year of indicator *j*.

Step 2: The entropy value of the annual indicators of each region of the Yangtze River Economic Belt is calculated, and the proportion of each index is obtained. The entropy calculation formula for item j indicator is as follows:


(5) $$h_{i}=-\frac{1}{1\mathrm{nm}}\overset{n}{\underset{i=1}{\sum }}p_{ij}1np_{ij}$$

Where *h_i_* represents the specific entropy value of indicator *j*.

Step 3: Calculate the difference coefficient for the indicator. For indicator *j*, the greater the *y_ij_* difference, the greater the impact on the results of specific provinces and cities, and vice versa. The specific calculation formula is as follows:


(6) $$f_{j}=1‐h_{j}$$

Step 4: Determine the weight *w_j_*. The calculation formula is as follows:


(7) $$w_{j}=\frac{f_{j}}{\sum_{j=1}^{n}\;}f_{j}$$

Step 5: Calculate the comprehensive measure value, that is, the calculation of the comprehensive score for the two systems of national fitness and national health. The calculation formula is as follows:


(8) $$u_{s}=\overset{m}{\underset{j=1}{\sum }}w_{j}y_{ij}$$

Where *u_s_* in Equation (8) is a comprehensive measure of national fitness and national health systems.

#### 3.1.3. Coupling coordination model

The coupling coordination model can organically combine factors from different domains or systems, forming a unified system framework. This systemic approach allows for a better capture of the complex relationships among interrelated factors and variables, thereby providing more accurate and comprehensive results and predictions. The coordinated advancement of the deep integration of national fitness and national health is a complex systemic project. Therefore, this study establishes a “coupling coordination” calculation model for the National Fitness and National Health systems in order to analyze and evaluate the interconnectedness and coordination between these two systems. The formula for calculating the coupling coordination of the National Fitness System and National Health System is as follows (27–31):


(9) $$c=\left[\frac{f\left(x\right)g\left(x\right)}{\left[\alpha f\left(x\right)+\beta g\left(x\right)\right]}\right]k$$

(10) $$\rho =\alpha f\left(x\right)\beta g\left(x\right)\text{,}$$

(11) $$R={\left(c\times p\right)}^{1/2}$$

Where *c* represents the coupling degree between the National Fitness System and National Health System. *f*(*x*) and *g*(*x*) are the comprehensive evaluation index of the National Fitness System and National Health System of provinces and cities in the Yangtze River Economic Belt, respectively, obtained by the entropy method. And, α and β represent the pending coefficient, and R represents the coupling coordination. This paper believes that the importance of the two systems is the same, therefore, both α and β take 0.5, that is, α = β = 0.5. *k* is the adjustment coefficient, which is equal to 2. c∈[0,1], when *c* = 1, it indicates that national fitness and national health are in a highly coupled state; when *c* = 0, it means that there is no correlation between national fitness and internal elements of national health, disorderly development. Similarly, R∈[0,1]. Coupling coordination levels are classified here (32–34), as detailed in Table 1.

**Table 1:** Coupling coordination grading criteria.

Coupling (C)	0–0.3	0.3–0.5	0.5–0.8	0.8–1.0
Grade	Low-level coupling	Antagonistic stage	Breaking-in stage	High-level coupling

Coupling coordination degree (R)	0–0.3	0.3–0.5	0.5–0.8	0.8–1.0
Coupling coordination grade	Low-coupling coordination	Medium-coupling coordination	High-coupling coordination	Utmost-coupling coordination

### 3.2. Indicator system and data sources

The two major systems of the National Fitness and National Health are more complex in the mutual influence of their respective elements, rather than a one-to-one linear relationship. Most scholars believe that research on the coupling coordination of complex systems with coupled interaction relationships should be conducted based on the comprehensive evaluation of multiple indicators of two systems (35). In order to reveal the degree of coordination between the development of the National Fitness System and National Health System, based on the analysis of the connotation of the correlation model between the National Fitness System and National Health System, 18 indicators are specifically selected from six dimensions, including the national fitness environment, national fitness participation, national fitness investment, health environment construction, health education promotion, and the development of healthy populations. Finally, a comprehensive development evaluation system for the integration of the National Fitness System and National Health System will be formed, as shown in Table 2. The similarity between the evaluation system and previous studies lies in both starting from the of the National Fitness System and National Health System and selecting relevant dimensions and indicators for evaluation. However, the difference lies in the fact that previous studies only selected indicators based on the composition of individual systems within national fitness and national health. In contrast, this study selects key elements and indicators of national fitness and national health from the perspective of their composition and relational models. This construction method can provide a more comprehensive reflection of the integrated development status of national fitness and national health, considering multiple perspectives and dimensions, thereby achieving a higher level of comprehensiveness and detail.

**Table 2:** Evaluation system for the integration development of the National Fitness System and National Health System.

Coupling system	Tier 1 Indicators	Tier 2 Indicators	Weight	Data Source	Type*
National Fitness System	National fitness environment	Parks in the city (Number)	0.132544	(36)	+
		Sports coach	0.162824	(37)	+
		Number of legal persons in the culture/sports/entertainment industry	0.113528	(38)	+
	National fitness participation	Retail revenue of cultural/sporting goods and equipment (100 million Yuan)	0.108876	(39)	+
		Operating income of legal entities in the cultural/sports/entertainment industry (100 million Yuan)	0.061045	(40)	+
		Number of employees in cultural/sports/entertainment institutions in the city	0.120398	(40)	+
	Investment in national fitness	Public welfare Fund expenditure of sports Lottery (ten thousand Yuan)	0.107941	(41)	+
		Investment in fixed assets of the whole society in culture/Sports/entertainment industry (100 million Yuan)	0.084064	(42)	+
		Local financial expenditure on culture/sports/media (100 million Yuan)	0.108775	(43)	+
National Health System	Healthy environment construction	Urban green coverage area (hectares)	0.120364	(44)	+
		Total investment in urban environmental infrastructure construction (100 million Yuan)	0.109801	(45)	+
		Number of beds in primary health care institutions	0.116094	(46)	+
	Health education promotion	Public health education activities (times)	0.123588	(47)	+
		Number of health education training participants (person-times)	0.146254	(47)	+
		Number of special education schools	0.098588	(48)	+
	Healthy population development	Average life expectancy (years)	0.096046	(49)	+
		Number of cases of Class A and B infectious diseases per 100,000 people	0.091271	(50)	−
		Number of inpatients in primary medical and health institutions (10,000)	0.097989	(51)	−

*“+” indicates a positive indicator, while “−” indicates a negative indicator.

Considering the availability, systematicness and accuracy of sample data, this paper uses the panel data of 18 evaluation indicators of 11 provinces and cities in the Yangtze River Economic Belt in China during the five years from 2016 to 2020 as the analysis object. The data of each indicator mainly comes from the China Statistical Yearbook, the China Statistical Yearbook of Tertiary Industry, the China Environmental Statistics Yearbook, the China Health Statistics Yearbook, the China Economic Census Yearbook, the official website of the Ministry of Finance, etc. This article selects 11 provinces and cities in the Yangtze River Economic Belt as the main research area for three reasons: (1) The Yangtze River Economic Belt spans the east and west, with a population and economic total exceeding 40% of the country, and there is comparative analysis of data results; (2) As a new region for the implementation of China’s new round of reform and opening up, it has global influence and is representative in data analysis. (3) Promoting the development of the Yangtze River Economic Belt is of great practical significance for the CPC Central Committee and the State Council to grasp and guide the New Normal of economic development, scientifically plan the new chess game of China’s economy, and promote the coordinated development of regional economy, which is conducive to the industrial structure and urbanization layout along the Yangtze River, and promote economic efficiency and value added. The raw data on National Fitness and Nation Health of 11 provinces and cities in the Yangtze River Economic Belt was supplied in Supplementary Tables S1 and S2, respectively.

## 4. Results and analysis

### 4.1. Comprehensive evaluation index for the coordinated development of the National Fitness System and National Health System

Table 3 shows the comprehensive evaluation index for the coordinated development of the National Fitness System and National Health System in the Yangtze River Economic Belt region of China, calculated based on the entropy method. It can be seen that in 2016, Jiangsu Province and Zhejiang Province had significant advantages in the development of national fitness compared to other provinces and cities, followed by Hunan, Sichuan, Shanghai, and Hubei. The top 6 countries in the development of national fitness in 2020 were Sichuan, Jiangsu, Zhejiang, Hunan, Jiangxi, and Hubei. Overall, Sichuan, Jiangsu, and Zhejiang have obvious advantages in the national fitness development index. This result is consistent with Bai’s (2021) (52) study that the advantages of the development of national fitness in Sichuan, Jiangsu, and Zhejiang in China are obvious. The implementation of the “National Fitness Implementation Plan (2016–2020)” in Jiangsu Province has achieved practical results. By 2020, the public service capacity and equalization level of national fitness have significantly improved. The health level of the population has remained at the forefront of the country. The role of the national fitness industry has become more prominent in the construction of a well-off society in all respects and the development of a “strong, prosperous, beautiful, and advanced” new Jiangsu (53). In 2016, Jiangsu and Zhejiang ranked among the top two in the comprehensive evaluation index of the National Health System, while Chongqing and Guizhou had the lowest comprehensive evaluation index of the National Health System. In 2020, the comprehensive evaluation index of the National Fitness System in Jiangsu Province still ranked first. The basic pattern of comprehensive evaluation of the National Health System has changed, with Sichuan Province’s index surpassing Zhejiang’s, which coincides with the research results of Zhang et al. (54). This study suggests that the proportion of personal health expenditure in total health expenses in Sichuan Province has decreased from 34.21% in 2013 to 27.87% in 2020. Under the condition that the *per capita* health cost is lower than the national average, the main health indicators of residents such as *per capita* life expectancy, infant mortality rate, and maternal mortality rate have been achieved, which are all better than the national average. Additionally, the data also supports the effectiveness of the implementation of the “Implementation Plan for Health Lifestyle Action of the Whole Population (2017–2025)” in Sichuan Province. By 2020, the health literacy level of residents in the province reached 20%, creating a collective action by the whole society and promoting a favorable atmosphere for the promotion and adoption of healthy lifestyle practices (55).

**Table 3:** Comprehensive evaluation indexes of the two major systems of 11 provinces and cities in China’s Yangtze River Economic Belt region in 2016 and 2020.

Region	2016		2020	
	National Fitness	National Health	National Fitness	National Health
Shanghai City	0.153490	0.304177	0.335971	0.351415
Jiangsu Province	0.455675	0.591390	0.822842	0.647932
Zhejiang Province	0.373533	0.504400	0.656830	0.526232
Anhui Province	0.133029	0.352991	0.272910	0.402219
Jiangxi Province	0.071029	0.389916	0.244404	0.455824
Hubei Province	0.153228	0.378735	0.411898	0.364752
Hunan Province	0.223076	0.419428	0.393817	0.497384
Chongqing City	0.086249	0.191457	0.203692	0.262405
Sichuan Province	0.218438	0.620262	0.478445	0.637685
Guizhou Province	0.019094	0.224423	0.101683	0.245942
Yunnan Province	0.137138	0.277403	0.296689	0.304884

### 4.2. Overall evolution features of the coupling between the National Fitness System and National Health System in China’s Yangtze River economic Belt

The coupling coordination formula can be used to calculate the coupling value and coordination values of the National Fitness System and National Health System in 11 provinces and cities of China’s Yangtze River Economic Belt from 2016 to 2020, as shown in Table 4. It can be seen that in terms of the coupling degree between the National Fitness System and National Health System, Jiangsu Province had the highest coupling degree in 2016, with a value of 0.991564. The National Fitness System and National Health System were in a high-level coupling stage, while Guizhou Province had the lowest coupling degree, with a value of 0.537635. The system of National Fitness and National Health were in a transitional stage. In 2020, Shanghai City had the highest coupling degree, with a value of 0.999747. The National Fitness System and National Health System was in a high-level coupling stage, while Guizhou Province also had the lowest coupling degree, but the value increased to 0.909829. The National Fitness System and National Health System reached a high-level coupling stage. From the perspective of coupling coordination degree, in 2016, Jiangsu Province had the highest coupling coordination dispatch, with a value of 0.720497, indicating that the National Fitness System and National Health System in Jiangsu Province is in a highly coupled and coordinated state. In 2020, the coupling coordination degree in Jiangsu Province was also the highest, with a value of 0.801997, indicating that the National Fitness System and National Health System in Jiangsu Province reached an extreme coupling coordination state. In 2016 and 2020, the coupling coordination degree in Guizhou Province was consistently the lowest, with values of 0.255855 and 0.397668, respectively. This result is consistent with the research findings of Wang (56), who stated that Jiangsu Province has consistently adhered to the national strategy of National Fitness, strengthened the construction of fitness venues and facilities, and continuously improved the public sports service system. The deep integration of the National Fitness Program and National Health Program has been achieved in Jiangsu (56). The two systems were in a low and moderate coupling coordination state. The average coupling degree and co-ordination degree among provinces and cities in 2020 were higher than those in 2016, indicating that there has been some progress in the coupling and coordinated development of the National Fitness Program and National Health Program. It also demonstrates that the comprehensive effect resulting from the coordination and mutual promotion of “National Fitness Plan (2016–2020)” and the relevant policies formulated by local governments at all levels can effectively promote facilitate the deep integration of the National Fitness Program and National Health Program (57).

**Table 4:** Calculated results of coupling coordination degree of the two major systems of 11 provinces and cities in China’s Yangtze River Economic Belt in 2016 and 2020.

Region	2016		2020	
	Coupling degree	Coupling coordination degree	Coupling degree	Coupling coordination degree
Shanghai City	0.944243	0.464838	0.999747	0.586179
Jiangsu Province	0.991564	0.720497	0.992903	0.854499
Zhejiang Province	0.988827	0.658834	0.993888	0.766756
Anhui Province	0.891725	0.465509	0.981486	0.575600
Jiangxi Province	0.722079	0.407946	0.953330	0.577732
Hubei Province	0.905703	0.490816	0.998155	0.622582
Hunan Province	0.952158	0.553067	0.993224	0.665268
Chongqing City	0.925460	0.358473	0.992034	0.480824
Sichuan Province	0.877758	0.606702	0.989770	0.743206
Guizhou Province	0.537635	0.255855	0.909829	0.397668
Yunnan Province	0.941016	0.441639	0.999907	0.548414

**Figure 2:**
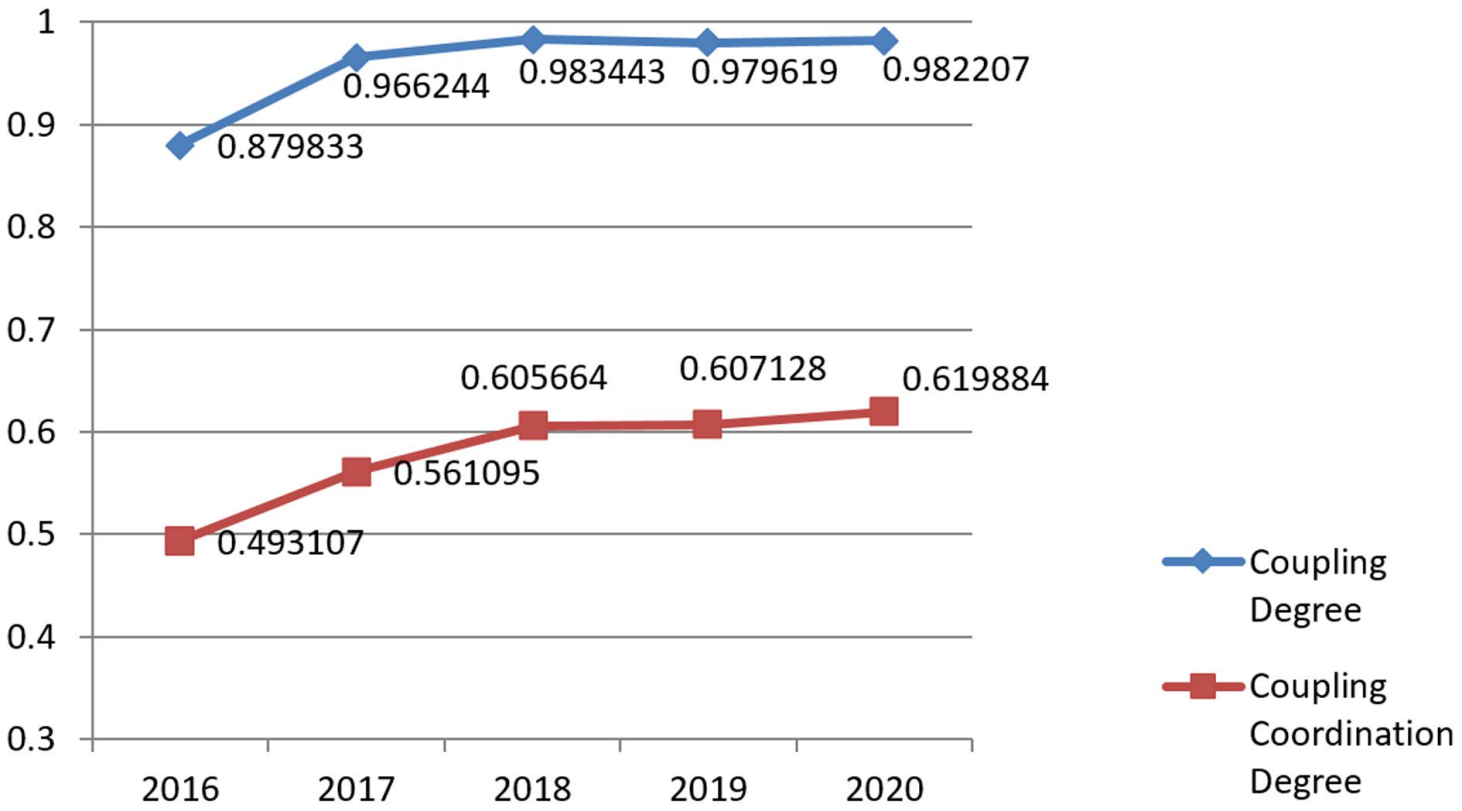
The mean value of the coupling coordination between the National Fitness System and National Health System in China’s Yangtze River Economic Belt.

The average coupling degree and coordination degree between the National Fitness System and National Health System in 11 provinces and cities of the Yangtze River Economic Belt in China are shown in Figure 2. The average coupling degree between the National Fitness System and National Health System from 2016 to 2020 was between 0.8–1.0, and overall, the National Fitness System and National Health System were in a high-level coupling stage. The average coupling coordination degree is between 0.4–0.7, and the coupling between the National Fitness System and National Health System is in a medium to high degree of coordination coupling. The fluctuation of the mean is not significant, reflecting the stability of the coupling coordination pattern of the two major systems. This result is also in line with Bao (58) use of national fitness to enhance the global influence of cities, reshape their image, promote urban regeneration, drive urban transformation, improve the health level of residents, and create a healthy city. This verifies the stable development and balanced promotion of the integration of the National Fitness System and National Health System.

### 4.3. Evolution of the coupling degree between the National Fitness System and National Health System in China’s Yangtze river economic belt

**Figure 3:**
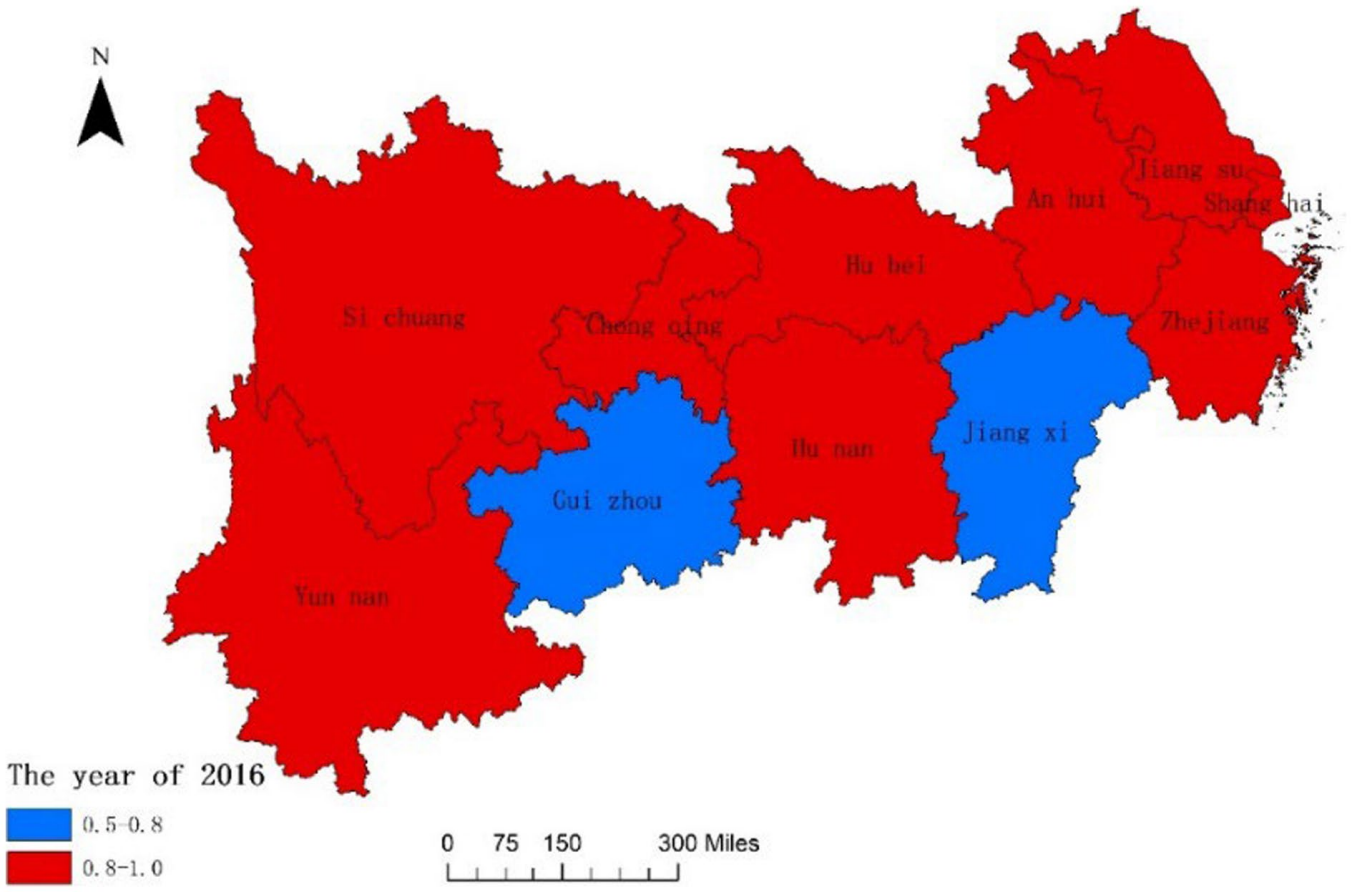
Spatial difference in coupling degrees of two major systems of 11 Provinces and Cities in China’s Yangtze River Economic Belt in 2016.

**Figure 4:**
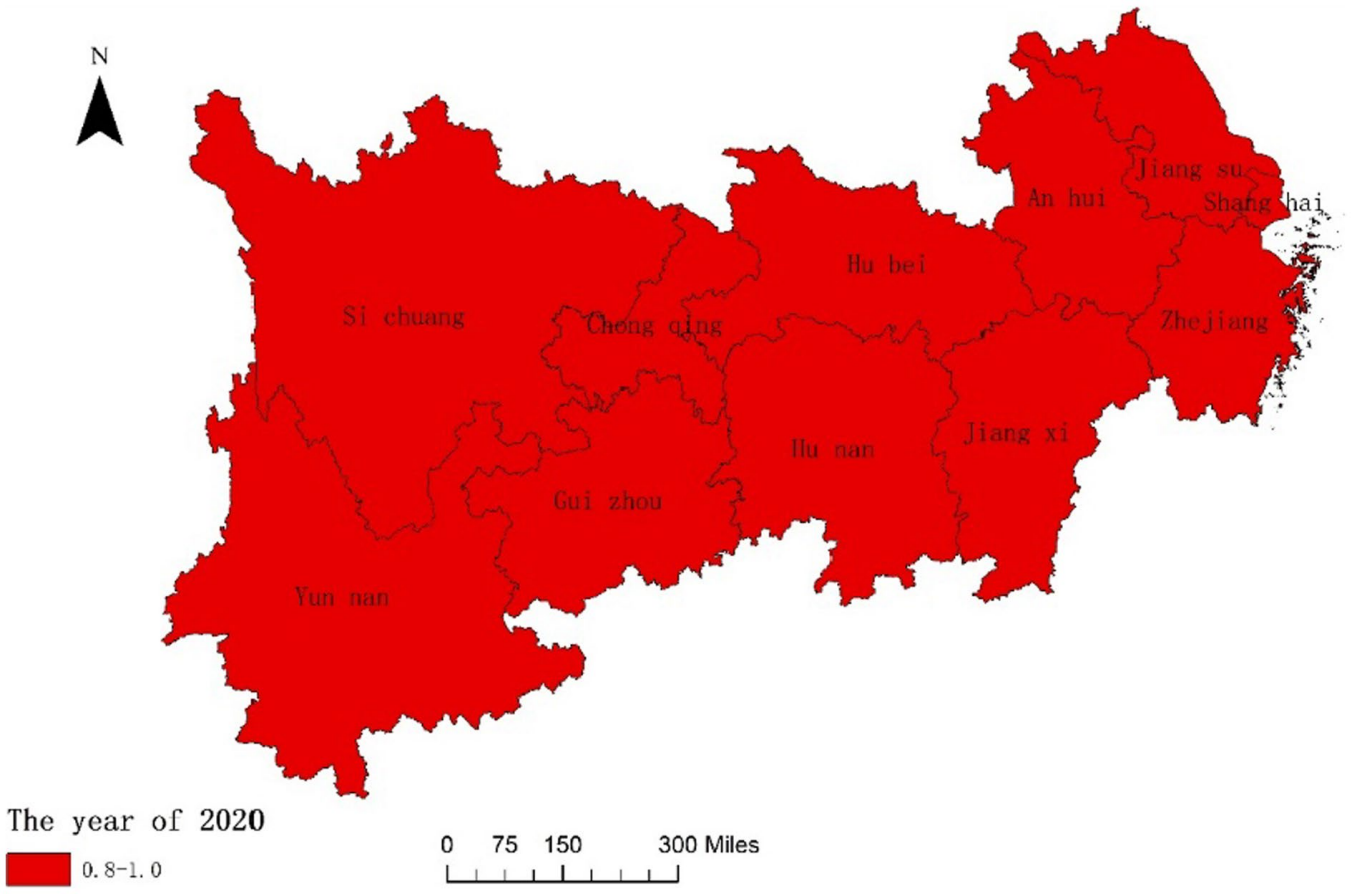
Spatial difference in coupling degrees of two major systems of 11 Provinces and Cities in China’s Yangtze River Economic Belt in 2020.

**Figure 5:**
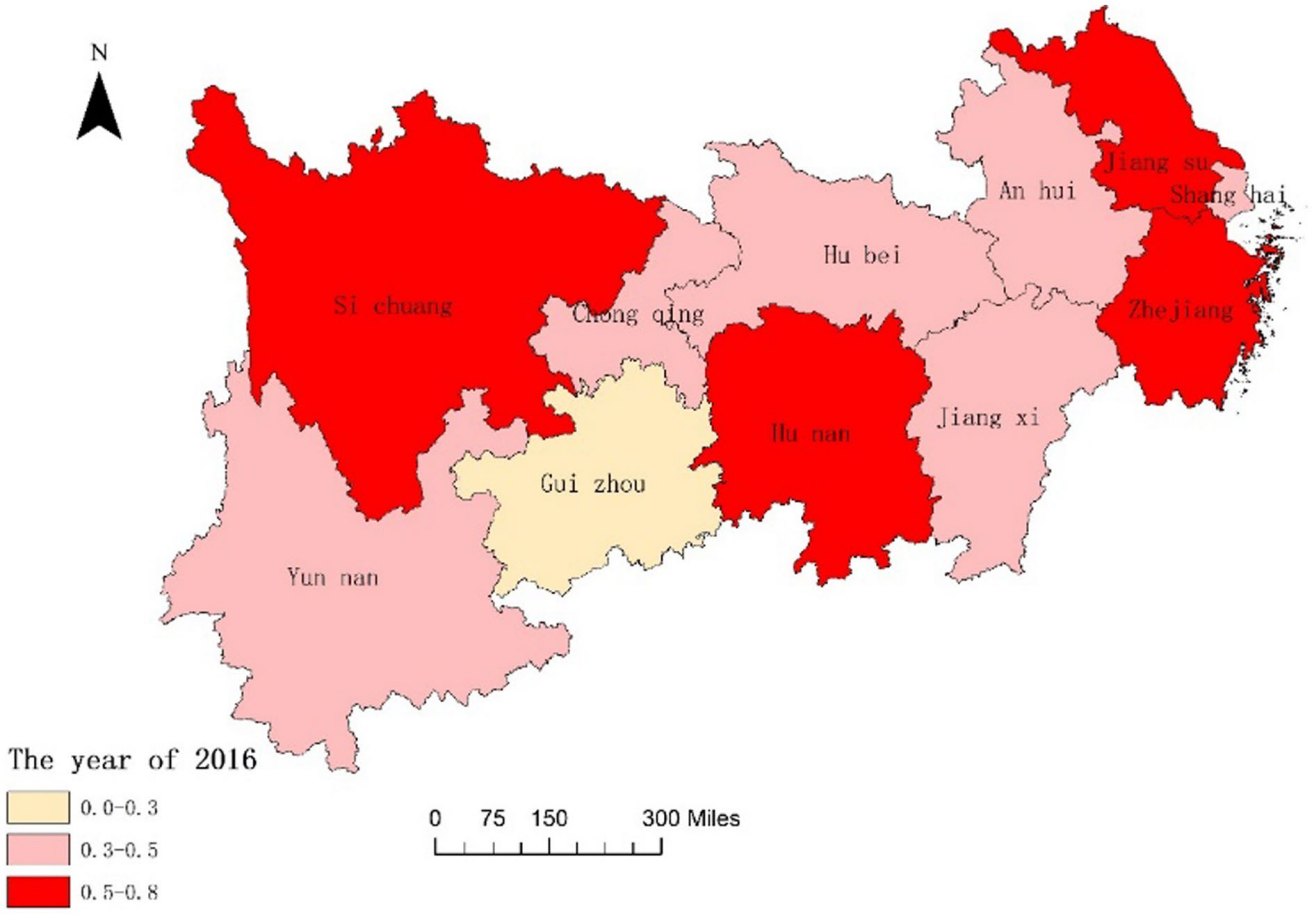
Spatial difference in coupling coordination degrees of two major systems in 11 Provinces and Cities of China’s Yangtze River Economic Belt in 2016.

**Figure 6:**
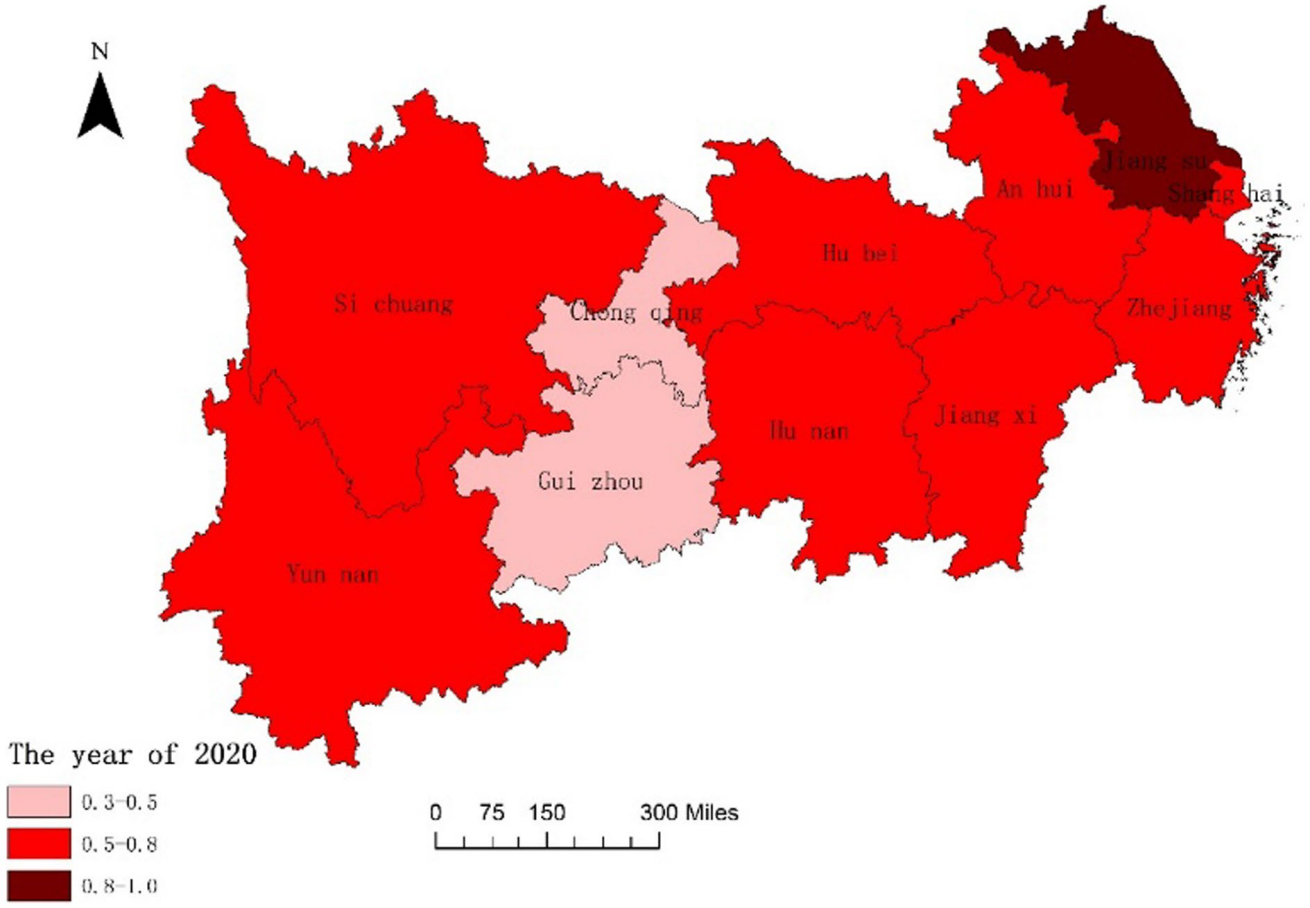
Spatial difference in coupling coordination degrees of two major systems in 11 Provinces and Cities of China’s Yangtze River Economic Belt in 2020.

In order to further analyze the dynamic changes of the coupling degree and coordination degree between the National Fitness System and National Health System in various provinces and cities of the Yangtze River Economic Belt in China over time and space, we combined ArcGIS reclassification tool to visualize the data, and the results are shown in Figures 3–6. From Figures 3, 4, it can be seen that in 2016, the provinces in the Yangtze River Economic Belt of China where the National Fitness System and National Health System was in a transitional stage were Jiangxi Province and Guizhou Province, while the other nine provinces and cities were in a high-level coupling stage. By 2020, the coupling degree of the 11 provinces and cities in the Yangtze River Economic Belt was in a high-level coupling stage, and the coupling degree of the two major systems of Jiangxi and Guizhou Provinces showed a significant increase from 2016 to 2020. This indicates that the two provinces have actively taken comprehensive measures to enhance the coupling and correlation between the National Fitness System and National Health System, in order to prevent the two systems from falling into an antagonistic phase. This result is consistent with the research results of Jiang (59) and Liu (60), which believe that in carrying out national fitness, Jiangxi and Guizhou provinces rely on resource endowments to design and develop characteristic fitness projects that endow folk customs, ancient post roads with red and other elements, seize the dividends of mass sports reform, adhere to the people-centered approach, and achieve health dividends and market dividends. By building a high-level public service system for national fitness, seizing the opportunity of assistance from the General Administration of Sport of China, promoting the participation of national fitness, effectively promoting the process of the Healthy Jiangxi and Healthy Guizhou actions, and promoting the deep integration of the National Fitness Program and National Health Program. Through the implementation of the “Jiangxi Province National Fitness Implementation Plan (2016–2020)” and the “Guizhou Province National Fitness Implementation Plan (2016–2020),” a high-level public service system for national fitness has been established. Seizing the opportunity provided by the support from the General Administration of Sport of China, these plans have encouraged the participation of the public in national fitness and effectively promoted the progress of the Healthy Jiangxi and Healthy Guizhou initiatives, facilitating the deep integration of the National Fitness Program and National Health Program (61, 62). It indicates that the integrated development of the National Fitness Program and National Health Program is positively influenced by economic level and regional marketization level.

### 4.4. Pattern evolution of the coupling coordination degree between the National Fitness System and National Health system in the China’s Yangtze River economic belt

The coupling coordination degree combines the coupling relationship between the National Fitness System and National Health System as a whole, which is more comprehensive and stable than the coupling results. The higher the coordination between the National Fitness System and National Health System, the higher the overall level of the two, and the greater the mutual promotion between the National Fitness System and National Health System. Conversely, one party hinders the development of the other party, forming a mutually contained and vicious cycle. From Figures 5, 6, it can be seen that in 2016, the province where the National Fitness System and National Health System was in a low degree of coupling and coordination was Guizhou Province. Although the coupling and coordination level of Guizhou Province improved in 2020, it is still in a moderate coupling and coordination state. Guizhou Province is located in the southwestern region of China, with beautiful mountains and rivers, pleasant climate, numerous ethnic groups, and abundant resources. However, its economic and educational development level is relatively backward. In recent years, sports and fitness venues in Guizhou Province have made great progress compared to before, but there is still a certain gap compared to developed provinces and cities. The lagging construction of sports and fitness facilities has to some extent hindered their coupling and coordination with the overall health of the people. Tong (63) believes that the advantages of the unbalanced development approach are clear development priorities, strong sequencing, and the ability to fully leverage the human, material, and financial resources advantages of each region, enabling the rapid development of a certain region’s sports and fitness industry in a relatively short period of time, while also forming a driving effect on other regions. Its weakness is that the gap in the development level of the sports and fitness industry among different regions is rapidly widening, which may cause psychological imbalance in areas with lower development levels, leading to a lag in the development of national fitness in the region, and even causing new problems. In 2016, the regions where the National Fitness System and National Health System was in a moderately coupled and coordinated state mainly included Shanghai City, Anhui Province, Jiangxi Province, Hubei Province, Chongqing City, and Yunnan Province. In 2020, Shanghai City, Anhui Province, Jiangxi Province, Hubei Province, and Yunnan Province transitioned from a moderately coupled and coordinated state to a highly coupled and coordinated state, while Chongqing’s coupled and coordinated state remained unchanged and remained moderately coupled and coordinated. In 2016, the regions where the National Fitness System and National Health System were in a highly coordinated and coupled state mainly included Jiangsu, Zhejiang, Hunan, Sichuan, etc. In 2020, Jiangsu Province reached an extremely coupled and coordinated state, and the highly coupled and coordinated regions have also expanded. Jiangsu Province is one of the most economically developed provinces in China, with a total GDP ranking second in the country for a long time and a *per capita* GDP ranking first in the country. It is a major economic province and also a major province in culture, sports, and health service industries. The development of the sports and fitness industry and the construction of healthy cities can basically form a virtuous cycle. This is closely related to Jiangsu Province continuously introducing new policies to promote the levels of the National Fitness System and National Health System. For example, the “Implementation Plan for Implementing the Outline of Building a Sports Power” issued by Jiangsu Province in 2020 explicitly states that by 2022, a new pattern of sports development will be formed, featuring strong government leadership, broad social participation, and a vibrant market. The physical literacy and health level of the population will continue to improve, new achievements will be made in sports reform and innovation, public services will be more balanced and sufficient, and the comprehensive strength of sports development will consistently rank among the top in the country, thus continuously increasing the contribution and influence of sports on economic and social development (64). Overall, most provinces and cities in the Yangtze River Economic Belt of China have a high degree of coupling and coordination between the National Fitness System and National Health System. It is verified that the higher the coordination between the National Fitness System and National Health System, the positive mutual promotion and mutually beneficial development of the two.

## 5. Conclusion

This study combines qualitative and quantitative analysis to analyze the integration relationship and mechanism of fitness for all and health for all. Taking the panel data of 11 provinces and cities in the Yangtze River Economic Belt of China from 2016 to 2020 as an example, the comprehensive evaluation systems of the two systems are, respectively, constructed, and the evaluation and analysis are conducted according to the coupling coordination degree. The main research conclusions are as follows:

Firstly, the National Fitness Program has improved the fitness environment, increased participation in fitness, and increased health investment, thereby promoting national health. The National Health Program has optimized environmental construction, health education, and healthy populations, thereby driving national fitness.

Secondly, between 2016 and 2020, in the Yangtze River Economic Belt of China, Jiangsu and Zhejiang provinces ranked among the top three in the evaluation index of the National Fitness System and National Health System, belonging to the typical double high type. However, Chongqing and Guizhou have the smallest comprehensive evaluation index, indicating that they belong to the double low type of provinces and cities. This confirms that the integrated development of the National Fitness Program and National Health Program is positively influenced by economic level and regional marketization level.

Thirdly, from 2016 to 2020, the average coupling degree between the National Fitness System and National Health System in the Yangtze River Economic Belt of China was between 0.8 and 1.0, which was in a high-level coupling stage. The average coupling coordination degree was between 0.4 and 0.7, which was in a medium to high degree of coordination coupling. This indicates that the coupling coordination pattern of the two systems has a certain degree of stability, and it also verifies that there is stable development and balanced promotion in the integration of the National Fitness Program and National Health Program.

Fourthly, the high-level coupling stage between the National Fitness System and National Health System in the Yangtze River Economic Belt of China has increased from 9 provinces and cities to 11 provinces and cities between 2016 and 2020. The degree of coupling coordination has expanded from 4 provinces and cities to 9 provinces and cities, with Jiangsu Province reaching an extremely coupled coordination state. The higher the coordination between the National Fitness Program and National Health Program, the mutual promotion and mutually beneficial development of the two.

The above results provide an important reference basis for the integrated and coordinated development of the National Fitness Program and National Health Program and the implementation of the Healthy China strategy.

## 6. Contributions and limitations

Contributions of this paper are as follows:

1. Based on the composition and relational model of the National Fitness System and National Health System, a comprehensive evaluation system for the coupling degree of the National Fitness Program and National Health Program is constructed, which provides a beneficial supplement to the evaluation indicators of their coupling.
2. The establishment of the coupling coordination calculation model for the National Fitness Program and National Health Program provides a practical calculation model for evaluating their coupling degree, offering important reference for research and practice in related fields.
3. The statistical analysis of the coupling degree of the National Fitness Program and National Health Program in the Yangtze River Economic Belt of China reveals their integration mechanism, enriching the quantitative research on the integrated development of the National Fitness Program and National Health Program.

In this paper, the measurement of the coupling coordination relationship between the National Fitness System and National Health System is carried out on the basis of multi-index evaluation system, which overcomes the shortcomings of the incomplete single index representation system, but there are also limitations that the construction of the index system is relatively flexible, which may lead to certain deviations in the final evaluation results. In addition, an in-depth analysis of the influencing factors of the spatial differences in the coupling coordination relationship between the National Fitness System and National Health System in 11 provinces and cities in the Yangtze River Economic Belt has not been conducted in the paper, and the spatial differences in the coupling and coordination relationship between the two systems may be affected by factors such as location and transportation, social economy, resource endowment and infrastructure. Therefore, the internal mechanism of the spatial difference features of the coupling coordination relationship between the 11 provinces and cities of the Yangtze River Economic Belt in China needs to be further studied. Moreover, due to the difficulty in collecting the latest data of national fitness in China, the evaluation index system for the coupling of the National Fitness System and National Health System constructed at present does not fully reflect the latest development trends, but only reflects a trend, whose latest data need to be further mined in the subsequent research.

## Data availability statement

The original contributions presented in the study are included in the article/ Supplementary material, further inquiries can be directed to the corresponding author.

## Author contributions

CC contributions include writing original draft, investigation, and data curation. SY contributions include methodology, review, and editing. Both authors contributed to the article and approved the submitted version.
